# Reassortant Highly Pathogenic Influenza A(H5N6) Virus in Laos

**DOI:** 10.3201/eid2103.141488

**Published:** 2015-03

**Authors:** Frank Y.K. Wong, Phouvong Phommachanh, Wantanee Kalpravidh, Chintana Chanthavisouk, Jeffrey Gilbert, John Bingham, Kelly R. Davies, Julie Cooke, Debbie Eagles, Sithong Phiphakhavong, Songhua Shan, Vittoria Stevens, David T. Williams, Phachone Bounma, Bounkhouang Khambounheuang, Christopher Morrissy, Bounlom Douangngeun, Subhash Morzaria

**Affiliations:** Commonwealth Scientific and Industrial Research Organisation Australian Animal Health Laboratory, Geelong, Victoria, Australia (F.Y.K. Wong, J. Bingham, K.R. Davies, J. Cooke, D. Eagles, S. Shan, V. Stevens, D.T. Williams, C. Morrissy);; National Animal Health Laboratory, Vientiane, Laos (P. Phommachanh, B. Douangngeun);; Ministry of Agriculture and Forestry, Vientiane (S. Phiphakhavong, P. Bounma, B. Khambounheuang);; Food and Agricultural Organization of the United Nations Regional Office for Asia and the Pacific, Bangkok, Thailand (W. Kalpravidh, C. Chanthavisouk, J. Gilbert, S. Morzaria)

**Keywords:** influenza virus, viruses, highly pathogenic avian influenza, HPAI, H5N6 subtype, influenza, avian influenza, poultry, chickens, ducks, reassortant, Laos

## Abstract

In March 2014, avian influenza in poultry in Laos was caused by an emergent influenza A(H5N6) virus. Genetic analysis indicated that the virus had originated from reassortment of influenza A(H5N1) clade 2.3.2.1b, variant clade 2.3.4, and influenza A(H6N6) viruses that circulate broadly in duck populations in southern and eastern China.

Asian lineage influenza A(H5N1) viruses continue to cause serious disease in poultry and sporadic human infections (*1*). This disease was reported in 2004 in poultry in Laos that were infected with clade 1 influenza A(H5N1) virus and subsequently in poultry infected with clade 2.3.4 and 2.3.2 viruses in 2006 and 2008, respectively (*2*,*3*). Interclade reassortant influenza A(H5N1) virus genotypes homologous to viruses circulating in southern China and Vietnam have also been detected, which indicated previous transboundary virus transfers. However, influenza A(H5N1) virus in poultry has not been reported in Laos since mid-2010 (*4*). We report highly pathogenic avian influenza (HPAI) in poultry in Laos in March 2014 that was caused by an emergent reassortant influenza A(H5N6) virus, apparently imported by live poultry from China.

## The Study

Virus isolations were performed under Biosafety Level 3 containment. Animal trials were conducted after approval of the Australian Animal Health Laboratory Animal Ethics and Institutional Biosafety Committees.

**Figure 1:**
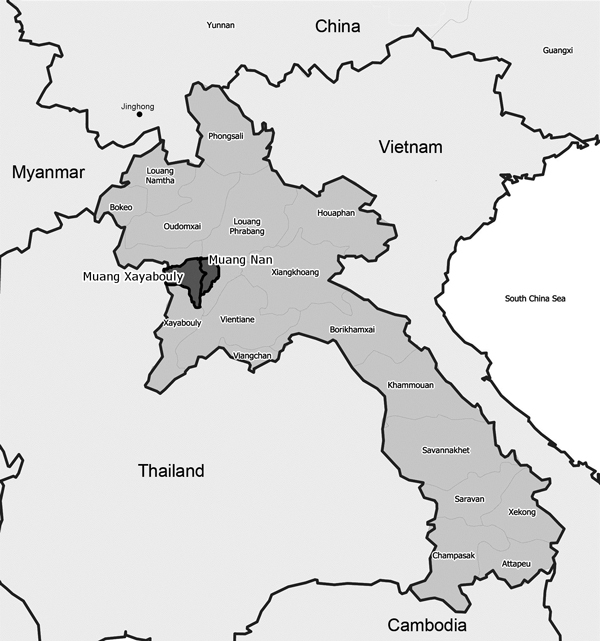
Locations of cases of highly pathogenic avian influenza in poultry caused by influenza A(H5N6) virus in Laos, March 2014. Dark gray shading indicates the 2 districts (Muang Nan and Muang Xayabouly) situated at the boundaries of Luang Prabang and Xayabouly Provinces, where villages with infected poultry were located. Affected birds were associated with regular consignments of mixed poultry transported from Jinghong and elsewhere in Yunnan Province, China.

After reports of disease in village poultry in Nan District, Luang Prabang Province, and Xayabouly District, Xayabouly Province (Figure 1), the Lao Provincial Agriculture and Forestry Office visited 2 villages during March 12–14, 2014, and collected samples from dead and sick birds for diagnosis. These birds were positive for avian influenza A virus (H5 subtype) by real-time reverse transcription PCR (RT-PCR) (*5*). Results were reported to the World Organisation for Animal Health on March 31, 2014 (*4*).

Infected poultry in both locations were 2 to 3–day-old chicks and ducklings imported from Jinghong in Yunnan Province, China, to a smallholder distributor in Luang Prabang on March 1. Consignments from this batch were delivered to the villages a week later, and birds at both locations showed clinical signs of influenza and died suddenly <24 h after arrival.

Respective pooled organ samples from a chicken and a duck from each village were sent to the Australian Animal Health Laboratory for analysis. These 4 samples were confirmed as positive for avian influenza virus (subtype H5) by RT-PCR but negative for neuraminidase (NA) subtype N1 and were subjected to virus propagation in 9 to 11–day-old specific pathogen–free chicken eggs.

Influenza genome sequencing was performed by using a MiSeq sequencer (Illumina, San Diego, CA, USA) and amplified avian influenza virus DNA libraries from a chicken sample from Luang Prabang and a duck sample from Xayabouly (*6*). Sequencing showed that an average of 98.6% reads mapped the virus genome with 210–481,069 coverage depth along different segments.

Hemagglutinin (HA) and NA genes were amplified from the 4 virus isolates by using RT-PCR and sequenced by using the Sanger method. Full-length HA and NA sequences from these isolates and 6 internal gene sequences from the 2 representative isolates shared 99%–100% nt identity, which indicated 1 influenza A(H5N6) virus genotype. Twenty consensus virus gene sequences were characterized and deposited in GenBank under accession nos. KM496962–KM496981.

Virus replicated in chicken eggs (titers >9 log_10_ 50% egg infectious doses). The 4 influenza A(H5N6) virus isolates were designated A/chicken/Laos/LPQ001/2014(H5N6), A/duck/Laos/LPQ002/2014(H5N6), A/chicken/Laos/XBY003/2014(H5N6), and A/duck/Laos/XBY004/2014(H5N6). Virus pathogenicity was evaluated by inoculation of six 4-week-old specific pathogen–free chickens with 6 log_10_ 50% egg infectious doses of A/duck/Laos/XBY004/2014(H5N6) from egg allantoic fluid by the oral–nasal–ocular route. 

Clinical signs, including facial swelling, hunching, fluffed feathers, depression, and huddling behavior, were observed in birds at 28 hours postinoculation. All birds were euthanized by 44 hours postinoculation for ethical reasons. The short incubation period, rapid progression of fulminant disease, and abundant viral antigen in multiple tissue and cell types were consistent with HPAI.

Analysis of each genome segment indicated that the Laos influenza A(H5N6) virus (LAO/14) is a novel triple reassortant. All genome segments of LAO/14 had highest (99%) GenBank sequence matches with corresponding genes of A/duck/Guangdong/GD01/2014(H5N6) (GD01/14), a virus independently identified in March 2014. We performed maximum-likelihood phylogenetic analysis on the 8 gene segments of LAO/14 by using the MEGA6 program (*7*) and avian influenza virus (subtypes H5 or N6) sequences from GenBank.

**Figure 2:**
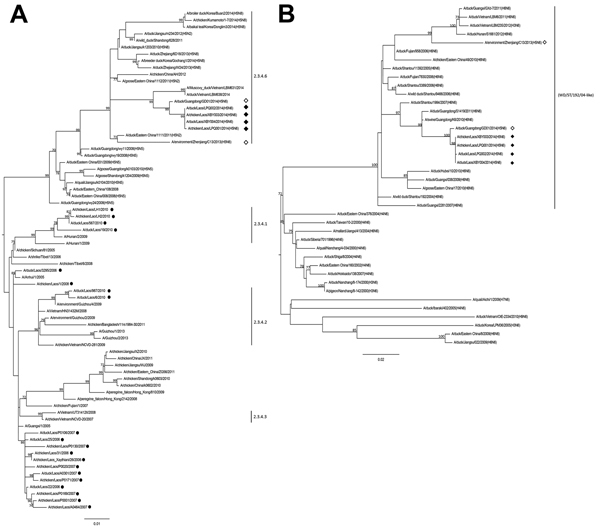
Phylogenetic analyses of influenza A(H5N6) viruses detected in Laos, March 2014, on the basis of the hemagglutinin (HA) and N6 neuraminidase (NA) genes. A) HA subtree showing relationships of emergent influenza A(H5N6) viruses with clade 2.3.4 H5 avian influenza viruses and B) NA subtree showing relationships with Asian lineage N6 avian influenza viruses. Vertical lines denote H5 subtype virus clades on the HA tree and the WD/ST/192/04 (A/wild duck/Shantou/192/2004)-like N6 gene pool on the NA tree. Proposed clade 2.3.4.6 has not been formally recognized by the World Health Organization/World Organisation for Animal Health/Food and Agricultural Organization of the United Nations H5N1 Evolution Working Group. Black diamonds indicate viruses identified in this study, white diamonds indicate Asian influenza A(H5N6) viruses identified in other studies, and black circles indicate viruses previously identified in Laos. All viruses are subtype H5N1 unless otherwise indicated. Bootstrap values ≥70% from 1,000 replicates are indicated at relevant nodes, and scale bars indicate nucleotide substitutions per site. The full HA and NA trees are provided in the online Technical Appendix Figure, panels A and B (http://wwwnc.cdc.gov/EID/article/21/3/14-1488-Techapp1.pdf).

HA gene phylogeny confirmed that LAO/14 and GD01/14 were closely related and belonged to clade 2.3.4.6, which was proposed for H5 subtype HPAI viruses with N1, N2, and N8 subtypes detected in poultry in China since 2010 (*8*) and in Vietnam in 2014 (Figure 2). The progenitor influenza A(H5N6) virus reassortant might have derived its HA gene from A/wild duck/Shandong/628/2011(H5N1)–like viruses in eastern China (Figure 2). Another virus, A/environment/Zhenjiang/C13/2013(H5N6), which has a similar genotype but independently reassorted gene lineages, had also been identified in Jiangsu Province (Figure 2; Technical Appendix). The same clade 2.3.4 H5 subtype virus donor pool resulted in a reassortant influenza A(H5N8) virus that has caused influenza outbreaks in poultry in South Korea since January 2014 (*9*).

Mature HA proteins of LAO/14 and GD01/14 have amino acids H103, N182, G221, Q222, and G224 (H5 numbering), which indicates that a preference for avian-like α2,3-sialic acid receptor binding is probably retained (*10*,*11*). Influenza A(H5N6) viruses have the HA cleavage sequence PLRERRRKR/GLF that is common in clade 2.3.4 HPAI viruses. Additional HA1 sites that might contribute to receptor binding and antigenic properties of LAO/14 are shown in Table 1.

**Table 1:** Amino acid substitutions in translated mature HA1 proteins of influenza A(H5N6) virus from Laos and H5 clade 2.3.4 reference viruses*

Mature HA1 position (H5 numbering)†	H5 subtype virus clade‡						
	DK/LAO/XBY4 (H5N6) 2.3.4.6	DK/GD/GD01 (H5N6) 2.3.4.6	WD/SD/628 (H5N1) 2.3.4.6	CK/LAO/XNY26 (H5N1) 2.3.4	JWE/HK/1038 (H5N1) 2.3.4	DK/LAO/3295 (H5N1) 2.3.4	ANH/1 (H5N1) 2.3.4
40	R	R	K	K	K	K	K
**45**	N	N	N	D	D	D	D
53	K	K	K	R	R	R	R
72	R	R	R	N	N	N	N
82	R	R	R	K	K	K	K
95	L	L	L	F	F	F	F
114	T	T	I	I	I	I	I
115	L	L	L	Q	Q	Q	Q
123	P	P	P	S	S	S	S
**124**	N	N	D	D	D	D	D
127	T	T	T	A	A	A	A
129	L	L	L	S	S	S	S
**133**	A	A	A	S	S	S	S
**140**	M	M	A	T	T	T	T
151	T	T	I	I	I	I	I
155	D	N	D	N	N	N	N
**156**	A	A	A	T	K	T	T
**162**	M	M	I	R	R	R	R
169	R	R	R	Q	Q	Q	Q
183	N	N	N	D	D	D	D
189	N	N	N	K	K	K	K
192	K	K	K	Q	Q	Q	Q
198	V	V	V	I	I	I	I
**210**	E	V	V	V	V	V	V
218	Q	Q	Q	K	K	K	K
223	R	R	R	S	S	S	S
240	H	H	H	N	N	N	N
**263**	T	T	T	A	A	A	A
265	M	M	M	M	M	I	V
269	M	M	V	V	V	V	V
273	H	H	H	N	N	N	N

*HA, hemagglutinin. †Sites associated with antigenicity are indicated in boldface (not comprehensive) (*11*). ‡Virus names: DK/LAO/XBY4, A/duck/Laos/XBY004/2014; DK/GD/GD01, A/duck/Guangdong/GD01/2014; WD/SD/628, A/wild duck/Shandong/628/2011; CK/LAO/XNY26, A/chicken/Laos/Xaythiani-26/2006; JWE/HK/1038, A/Japanese white-eye/Hong Kong/1038/2006; DK/LAO/3295, A/duck/Laos/3295/2006; ANH/1, A/Anhui/1/2005. Clade designations provided by the World Health Organization/World Organisation for Animal Health/Food and Agricultural Organization of the United Nations H5N1 Evolution Working Group unified nomenclature system for highly pathogenic avian influenza (H5N1) viruses (http://www.who.int/influenza/gisrs_laboratory/h5n1_nomenclature/en/). Proposed clade 2.3.4.6 has not been formally recognized.

The LAO/14 NA gene likely originated from group II lineage influenza A(H6N6) viruses that are established in domestic ducks in China (*12*) and have the highest (98%) nt identities with influenza A(H6N6) viruses isolated from domestic pig and live market poultry (Figure 2). LAO/14 and GD/14 influenza A(H5N6) viruses have the 11-aa deletion in the NA stalk region (positions 59–69; N6 numbering) found in influenza A(H6N6) viruses in China (*12*). Key known NA and matrix 2 inhibitor resistance markers were not observed in LAO/14 (*2*).

Influenza A(H5N6) viruses have an internal gene backbone from clade 2.3.2.1b influenza A(H5N1) virus, which is also found in domestic ducks from south-central and eastern China (*13*,*14*). The 6 internal genes of LAO/14 had highest (98%–99%) sequence matches with those of A/duck/Hunan/S4220/2011(H5N1) or A/duck/Zhejiang/2248/2011(H5N1). The polymerase basic 2 E627K mutation linked to mammalian host adaptation was not present in influenza A(H5N6) viruses (*10*). Phylogenetic trees of virus internal genes are shown in the Technical Appendix Figure, panels C–H. The influenza A(H5N6) virus from Zhenjiang, Jiangsu Province, China, had a nontruncated N6 NA and divergent A(H5N1) polymerase basic 2 gene lineage, which supports an independent reassortment origin.

Hemagglutination by LAO/14 was generally uninhibited by chicken or ferret antisera against reference influenza A(H5N1) virus, including antisera to a clade 2.3.4 virus from Laos (Table 2), by hemagglutination inhibition test. Antigenic divergence of LAO/14 from clade 2.3.4 viruses was supported by accumulation of 31-aa substitutions in their mature HA1 (Table 1). However, chicken antiserum against A/duck/Laos/XBY004/2014(H5N6) showed broader cross-reactivity with some viruses of other influenza A(H5N1) clades, which might indicate some conservation of epitopes.

**Table 2:** Hemagglutination inhibition assay of influenza A(H5N6) virus from Laos with chicken and ferret antisera against reference influenza A(H5N1) viruses*

Antigen	H5 subtype clade†	Reference chicken antiserum‡

CK/VNM/8 (H5N1) 1	CK/IND/BBVM204 (H5N1) 2.1.3	PH/VNM/3773 (H5N1) 2.3.2.1c	CK/LAO/XNY26 (H5N1) 2.3.4	CK/MYM/295 (H5N1) 2.3.2.1a	DK/LAO/XBY4 (H5N6) 2.3.4.6	CK NEG
Reference								
CK/VNM/8	1	**640**	20	40	80	80	80	<10
CK/IND/BBVM204	2.1.3	10	**1,280**	20	10	20	20	<10
PH/VNM/3773	2.3.2.1c	80	80	**640**	80	320	320	<10
CK/LAO/XNY26	2.3.4	160	80	40	**320**	160	80	<10
CK/MYM/295	2.3.2.1a	80	40	160	20	**320**	20	<10
Test								
CK/LAO/LPQ1	2.3.4.6	<10	<10	<10	<10	<10	320	<10
DK/LAO/LPQ2	2.3.4.6	<10	<10	<10	<10	<10	320	<10
CK/LAO/XBY3	2.3.4.6	<10	<10	<10	<10	<10	320	<10
DK/LAO/XBY4	2.3.4.6	40	<10	10	<10	<10	**640**	<10
		Reference ferret antiserum‡						
		JWE/HK/1038 (H5N1) 2.3.4	DK/LAO/3295 (H5N1) 2.3.4	ANH/1 (H5N1) 2.3.4	CM/HK/5052 (H5N1) 2.3.2.1	BS/HK/1161 (H5N1) 2.3.2.1b	HUB/1 (H5N1) 2.3.2.1a	DK/VNM/2848 (H5N1) 2.3.2.1c
Reference								
JWE/HK/1038	2.3.4	**80**	80	80	<40	<40	ND	ND
DK/LAO/3295	2.3.4	<40	**160**	80	<40	<40	<40	<40
ANH/1	2.3.4	<40	320	**320**	<40	<40	<40	<40
CM/HK/5052	2.3.2.1	<40	40	80	**160**	<40	<40	80
BS/HK/1161	2.3.2.1b	<40	<40	80	80	**320**	<40	80
HUB/1	2.3.2.1a	<40	<40	40	80	<40	**80**	40
DK/VNM/2848	2.3.2.1c	<40	<40	<40	40	160	<40	**160**
Test								
CK/LAO/LPQ1	2.3.4.6	<40	<40	<40	<40	<40	<40	<40
DK/LAO/LPQ2	2.3.4.6	<40	<40	<40	<40	<40	<40	<40
CK/LAO/XBY3	2.3.4.6	<40	<40	<40	<40	<40	<40	<40
DK/LAO/XBY4	2.3.4.6	<40	<40	40	<40	<40	<40	<40

*Virus names: CK/VNM/8, A/chicken/Vietnam/8/2004; CK/IND/BBVM204, A/chicken/Indonesia/ BBVM204/2007; PH/VNM/3773, A/pheasant/Vietnam/3773/2013; CK/LAO/XNY26, A/chicken/Laos/Xaythiani-26/2006; CK/MYM/295, A/chicken/Myanmar/295/2010; JWE/HK/1038, A/Japanese white-eye/Hong Kong/1038/2006; DK/LAO/3295, A/duck/Laos/3295/2006; ANH/1, A/Anhui/1/2005; CM/HK/5052, A/common magpie/Hong Kong/5052/2007; BS/HK/1161, A/barn swallow/Hong Kong/1161/2010; HUB/1, A/Hubei/1/2010; DK/VNM/2848, A/duck/Vietnam/NCVD-2848/2013; CK/LAO/LPQ1, A/chicken/Laos/LPQ001/2014; DK/LAO/LPQ2, A/duck/Laos/LPQ002/2014; CK/LAO/XBY3, A/chicken/Laos/XBY003/2014; DK/LAO/XBY4, A/duck/Laos/XBY004/2014; CK NEG, negative control chicken serum. †Clade designations provided by the World Health Organization/World Organisation for Animal Health/Food and Agricultural Organization of the United Nations H5N1 Evolution Working Group unified nomenclature system for highly pathogenic avian influenza (H5N1) viruses (http://www.who.int/influenza/gisrs_laboratory/h5n1_nomenclature/en/). Proposed clade 2.3.4.6 has not been formally recognized. ‡Values are titers. Homologous titers of reference virus antigen to its corresponding antiserum are indicated in boldface.

## Conclusions

After an absence of 4 years, HPAI in poultry in Laos was shown to be caused by an emergent reassortant influenza A(H5N6) virus. Genetic evidence indicates that this virus probably originated from domestic poultry in China. The common progenitor of LAO/14 and GD01/14 appears to have originated from reassortment of H5 clade 2.3.2.1b, H5 clade 2.3.4.6, and influenza A(H6N6) viruses that circulate in ducks in southern and eastern China. Coincidentally, the first fatal human infection with an influenza A(H5N6) virus in Sichuan Province was reported in May 2014 (*15*). Infection with influenza A(H5N6) virus was confirmed in poultry in Sichuan Province at that time (*4*), although the relationship of this virus with LAO/14 is unclear.

Influenza A(H5N6) viruses might already be widely distributed; poultry in Vietnam have been affected since April 2014 (*4*). LAO/14 was antigenically distant to clade 2.3.4 viruses, which raises concerns about effectiveness of current poultry vaccines against this virus, as well as vaccine candidate selection for prepandemic preparedness.

**Technical Appendix.** Phylogenetic analyses of influenza A(H5N6) virus detected in Laos, March 2014, on the basis of the 8 influenza virus genes.
