# Supplementary material for: Reassortant Highly Pathogenic Influenza A(H5N6) Virus in Laos
Source: Emerg Infect Dis. 2015 Mar;21(3):511–6. doi: 10.3201/eid2103.141488 (PMC4344285; doi:10.3201/eid2103.141488)
Supplement: Supplementary file 1 — Technical Appendix. Phylogenetic analyses of influenza A(H5N6) virus detected in Laos, March 2014, on the basis of the 8 influenza virus genes. [file 14-1488-Techapp-s1.pdf]

DOI article: http://dx.doi.org/10.3201/eid2103.141488

# Reassortant Influenza A(H5N6) Virus in Laos

**Technical Appendix. Phylogenetic analyses of influenza A(H5N6) virus detected in Laos, March 2014, on the basis of the 8 influenza virus genes.**

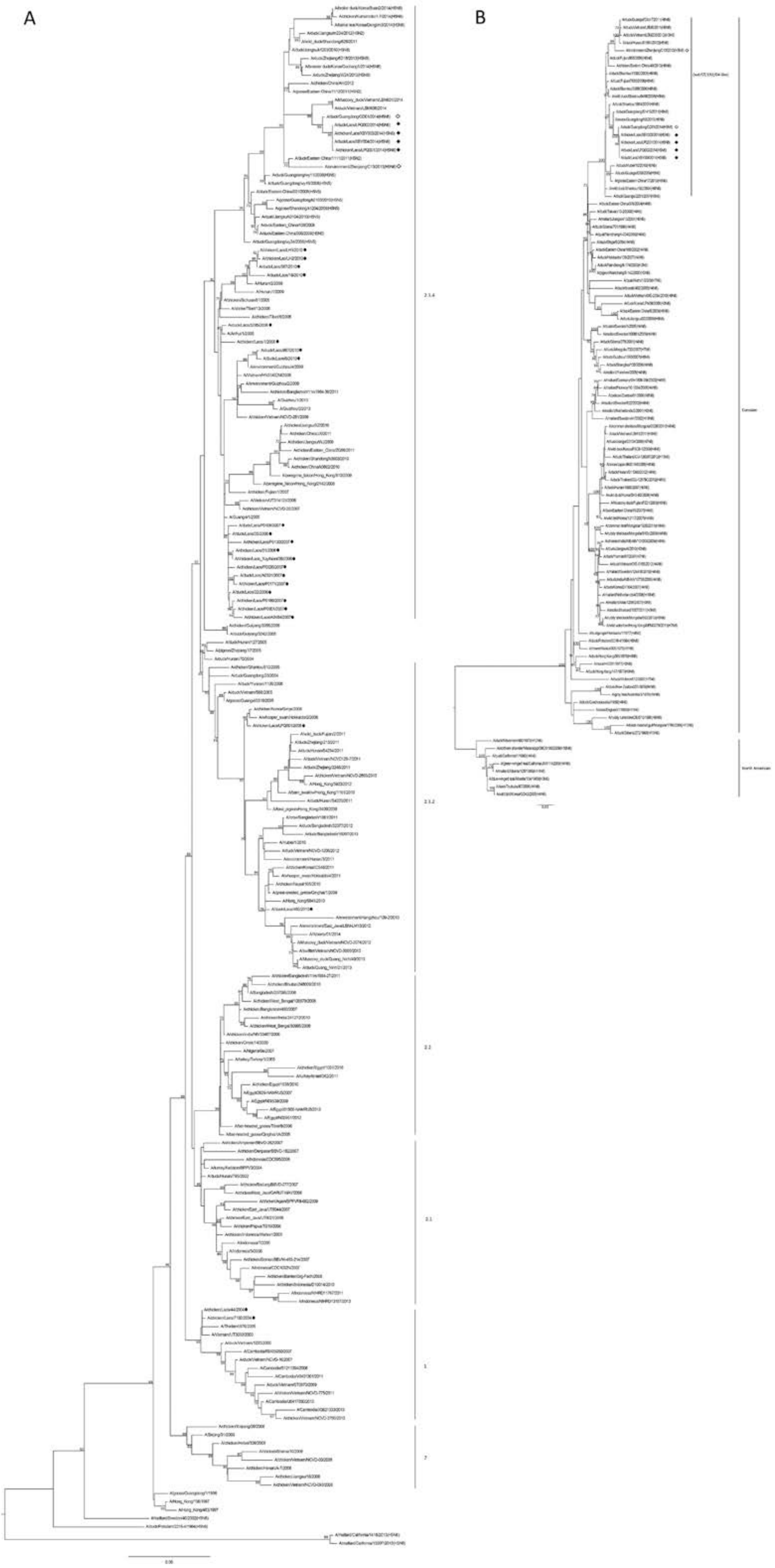

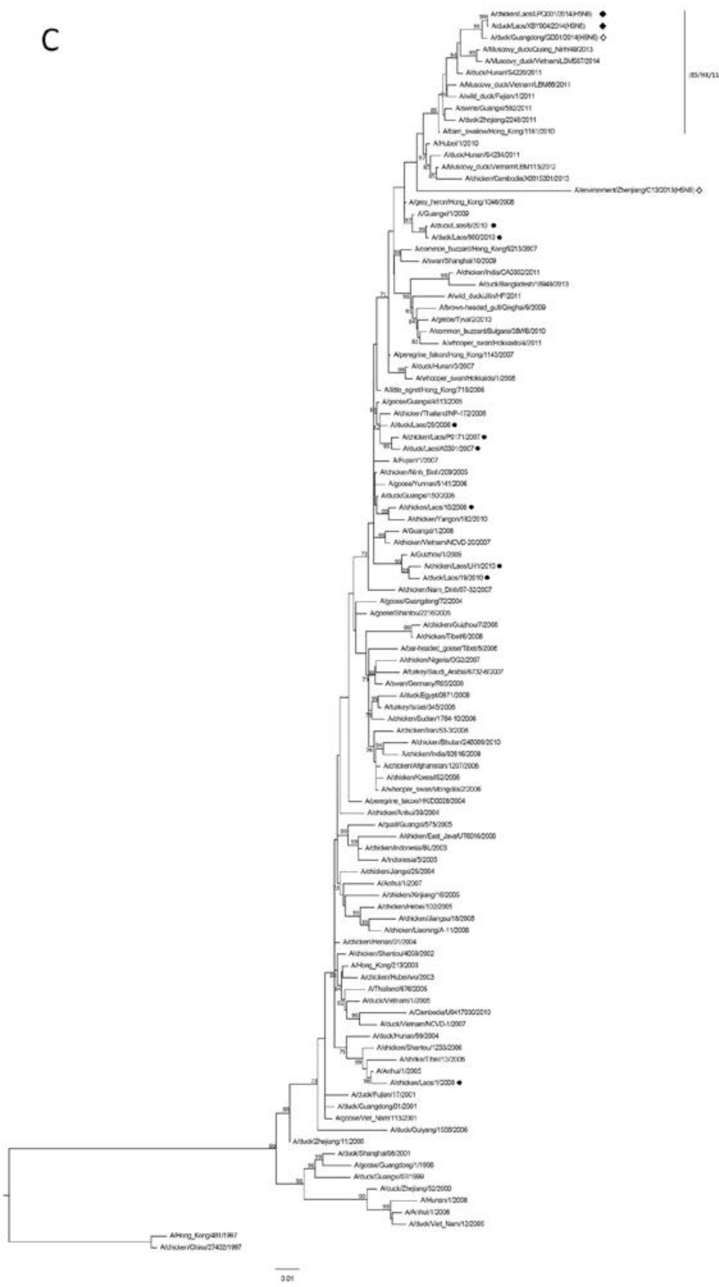
C

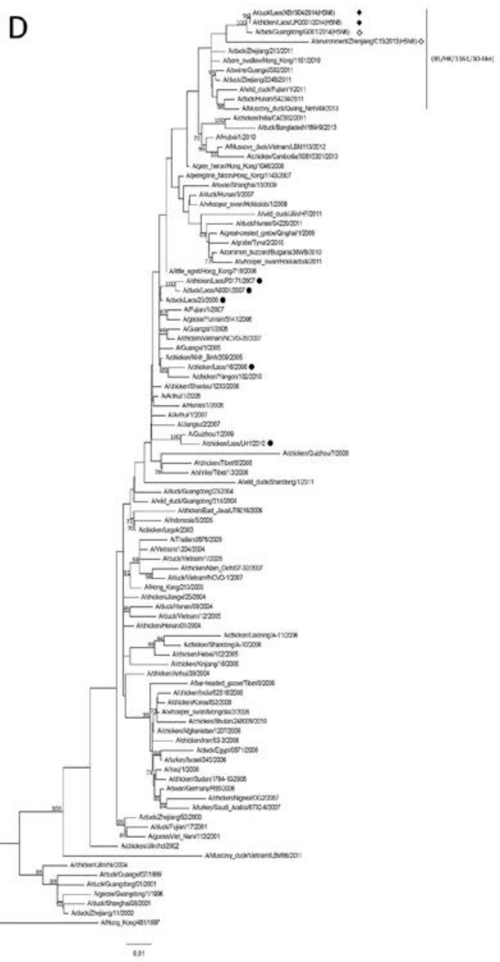
D

E

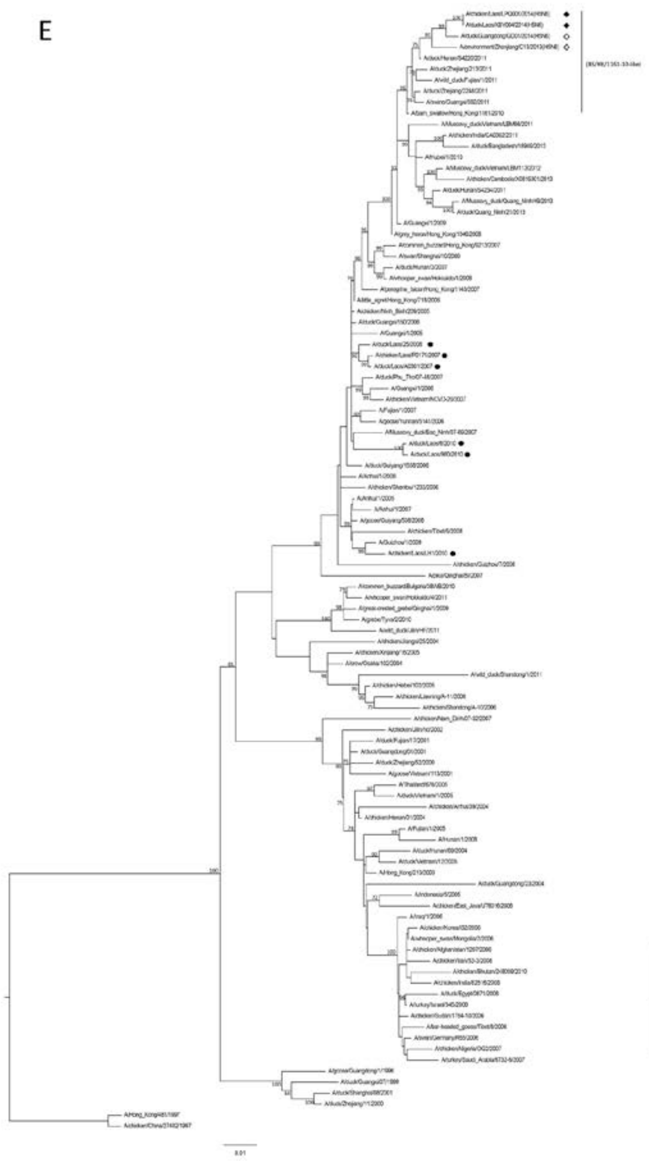

F

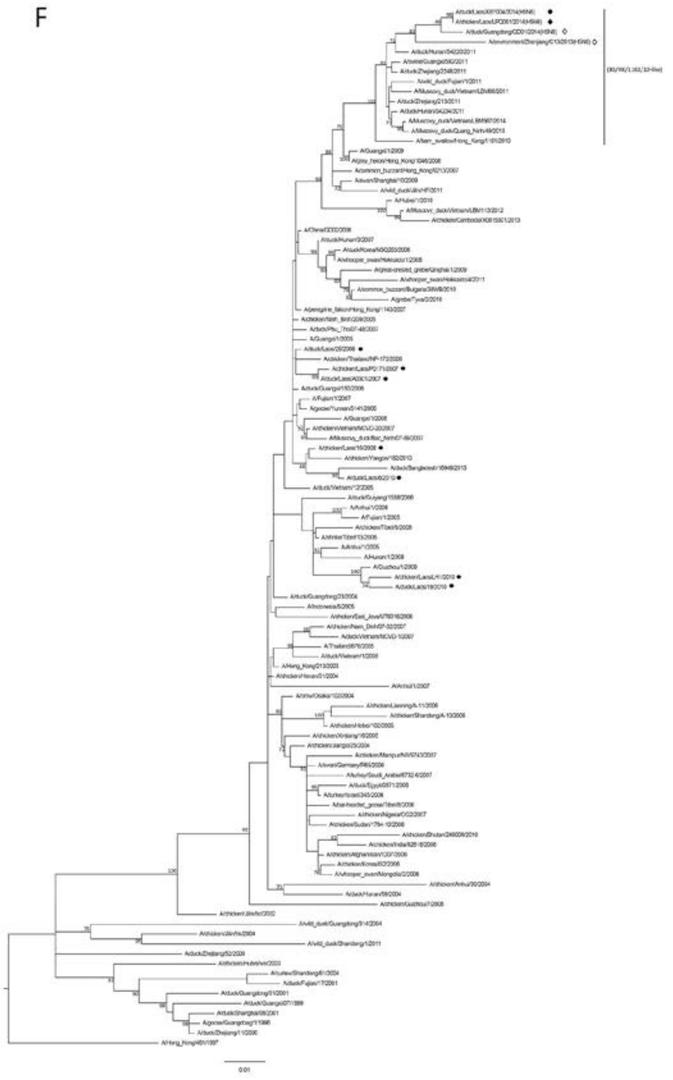

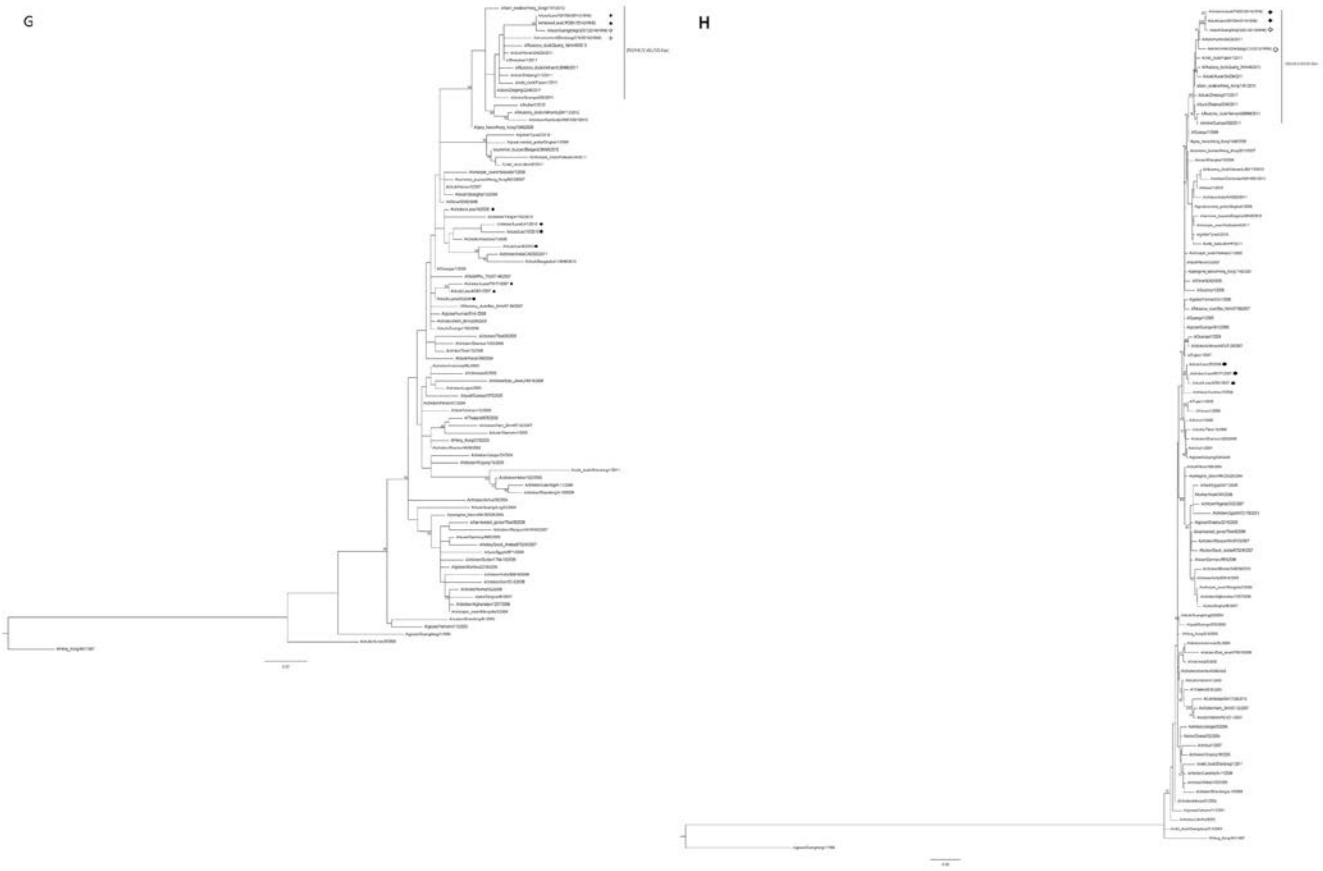


**Technical Appendix Figure.** Phylogenetic analyses of influenza A(H5N6) virus detected in Laos, March 2014, on the basis of the 8 influenza virus genes: A) HA; B) NA; C) PB2; D) PB1, E) PA; F) NP; G) MP; and H) NS. Vertical lines denote H5 subtype virus clades on the HA tree (panel A), major N6 virus lineages and the WD/ST/192/04 (A/wild duck/Shantou/192/2004)-like N6 gene pool on the NA tree (panel B), and the BS/HK/1161/10 (A/barn swallow/Hong Kong/1161/2010)-like (clade 2.3.2.1b) A(H5N1) gene pool on the 6 internal gene trees (panels C–H). Black diamonds indicate viruses identified in this study, white diamonds indicate Asian influenza A(H5N6) viruses identified in other studies, and black circles indicate viruses previously identified in Laos. All viruses are subtype H5N1 unless otherwise indicated. Maximum-likelihood phylogenetic trees were estimated by using the best-fit general time reversible base substitution model with gamma distributed rate variation and invariant sites. Trees were constructed from 185 HA sequences of 1,548 nt, 102 N6 NA sequences of 1,413 nt, 106 PB2 sequences of 2,262 nt, 96 PB1 sequences of 2,271 nt, 102 PA sequences of 2,148 nt, 105 NP sequences of 1,494 nt, 87 MP sequences of 979 nt, and 91 NS sequences of 69 nt. The HA and NA trees were rooted to outgroup North American H5N6 and N6 subtype lineage branches, respectively; the PB2, PB1, PA, NP, and M trees were rooted to the Hong Kong/97 lineage branch; and the NS tree was rooted to the goose/Guangdong/96 branch. Bootstrap values ≥70% from 1,000 replicates are indicated at relevant nodes. Scale bars indicate nucleotide substitutions per site. HA, hemagglutinin; NA, neuraminidase; PB, polymerase basic; PA, polymerase acidic; NP, nucleocapsid protein; MP, matrix protein; NS, nonstructural.
